# Preparing Undergraduates for the Global Future of Health Care

**DOI:** 10.5334/aogh.2456

**Published:** 2019-03-19

**Authors:** Roxanne Amerson

**Affiliations:** 1 Clemson University, Greenville, US

## Abstract

**Background:**

Health professionals must be academically and experientially prepared regarding the social determinants of health to reduce health disparities at the global level. The emerging literature reflects a trend for incorporating global health competencies for health care. Specifically, recommendations from the Consortium of Universities for Global Health, National Academy of Medicine, and multiple nursing organizations encourage the inclusion of a global health curriculum.

**Objectives:**

To describe the development of an undergraduate global health certificate program and provide recommendations for the development of future global health programs.

**Findings:**

At the completion of the certificate program, students felt better prepared to apply course content to culturally diverse populations in low resource settings.

**Recommendations:**

Before developing a global health program, preconceived ideas about study abroad experiences and faculty concerns associated with course overload should be dispelled through evidence-based, educational sessions. Curricular time constraints in content-laden programs should be mitigated through an appropriate mix of e-learning formats. Last, a strong value must be placed on interprofessional education to facilitate capacity building through a bi-directional flow of knowledge and resources between the educational institution and the host country.

## Introduction

Global health opportunities to work in low resource countries or settings and the influx of immigrant populations necessitate that health professionals be prepared to deliver health care with a global perspective; yet, the content-laden programs of health-related disciplines frequently have a limited curriculum related to global health. In 2010, curriculum guidelines for family medicine residents called for the inclusion of global health competencies, incorporating knowledge, attitudes, and skills for working in low resource settings [1]. A collaborative task force with the American Academy of Nursing Expert Panel on Global Nursing and Health and the Transcultural Nursing Society issued guidelines for implementing culturally competent nursing care [2], which included the need for formal education and clinical training as part of a global agenda. A report from the National Academy of Medicine (NAM) [3] emphasized the crucial need for all health profession students to be educated about the social determinants of health and the impact on care for underserved populations. Also, the Council for the Advancement of Nursing Science recommended priorities for nursing science to address global health issues and nursing care in under-resourced countries [4]. Furthermore, a call to action for nursing encouraged a “global innovation flow” between global partners (p. 2) [5]. This flow of information should be bi-directional with shared responsibilities between high resource and under-resourced countries to meet the challenges of global health [6]. Premji and Hatfield [5] direct attention to the “One World, One Health” paradigm, which emphasized the need for interconnectedness and partnerships to address health needs without being hampered by borders. International collaboration allows the transfer of shared knowledge through research efforts to improve health outcomes within and across borders, while taking into consideration that those who engage in research in under-resourced countries must be cognizant of the unique cultural and ethical challenges of conducting research at the international level. The premier nursing organization, Sigma Theta Tau International (STTI), further emphasizes this growing trend through the organization’s global initiatives. In 2015, the Consortium of Universities for Global Health (CUGH) published a set of proposed interprofessional global health competencies to provide consistency across programs [7]. Most recently, McDermott-Levy, Leffers, and Mayaka [8] provided ethical principles and guidelines for global health nursing practice in low to middle resource countries. It is clear from the increased focus on global health and the proposed competencies to deliver care in low resource settings that academic programs need to provide opportunities for in-depth knowledge, increased skills, and reflective attitudes to address the evolving health needs of the global community.

To promote improved global health outcomes while providing culturally competent care, completion of a global health certificate program (GHCP) provides one avenue to prepare students to work in low resource countries or settings around the world. The purpose of this paper is to describe the development of an undergraduate global health certificate program and provide recommendations for the development of future global health programs.

## Background

Numerous nursing programs throughout the US engage students in global health experiences through international service-learning or immersion experiences in foreign countries [6,9,10,11,12]. Unfortunately, many departments within universities provide embedded study abroad (SA) programs (experiences which are embedded within a discipline-specific course). While this is an effective approach for the individual department, it encourages working in “silos” and limits students’ exposure to the role of interdisciplinary teams required for successful global work. Health care policy advocates urge educators to reject this mentality and negotiate workloads that are distributed among faculty of related disciplines [13]. Currently, few best practices exist to provide models for this type of interdisciplinary education; thus, this unique program will serve as a model for other institutions. While the GHCP is spearheaded by a School of Nursing, faculty from other disciplines are encouraged to participate in the SA component. This shared responsibility has the potential to decrease fiscal burdens by sharing the course workload associated with implementing a SA course, while providing interprofessional education.

The health comptencies proposed by CUGH further emphasize the importance of interprofessional collaboration for the field of global health [7]. The NAM Report on Social Determinants also accentuates the need for interprofessional, experiential learning [3]. According to Meleis [14], NAM issued recommendations for transformative education which emphasizes the importance of interprofessional education, but barriers to this implementation still exist in the current educational environment of nursing and medicine as well as other disciplines. One barrier to interprofessional education is the rigidity of curricula for science, technology, engineering and mathematics (STEM) majors, which leaves little room for inclusion of SA. These barriers are removed by making SA more relevant and accessible to these underrepresented fields of study. Global health programs must facilitate academic flexibility and articulate a pathway with relevance for various degree programs; thereby facilitating interprofessional education.

The vast majority of global health programs are offered at the graduate level; with few options for global health certificates available to undergraduates. Equipped with knowledge of the aforementioned challenges, the recommendations of NAM, the strategies for mapping global health competencies for undergraduate curriculum [15], and the CUGH global health competencies; the faculty embarked on a mission to develop a curriculum for an undergraduate GHCP.

## Curriculum Planning and Development

A core group of faculty with clinical expertise in community health, formal training in international public health, and a decade of experience in leading SA trips to low-resource countries, began the process of developing a curriculum. Based on the CUGH global health competencies, Tier 1 Quad Council Public Health Nursing Competencies [16], and recommendations for implementing international service-learning [9], the faculty identified the program outcomes, the course objectives, and the rationale for implementation of this new program. Certificate programs typically range from nine credit hours to 24 credits depending on the individual university’s policies. Keeping in mind the heavy curricular loads of nursing and other health-related disciplines, a curriculum was developed with 12-credit hours; making the curriculum feasible, yet sufficient to provide knowledge at the undergraduate level for entry-level work in low resource settings. The curriculum incorporated the total CUGH competencies for the global citizen level (Level I) and the majority of the competencies for the basic operational program-oriented level (Level III). The global citizen competencies are applicable for all disciplines; while the program-oriented operational level competencies are applicable for specific disciplines such as nursing or medicine [7]. Development of the Level II and Level IV competencies by CUGH is ongoing at present. Dawson et al [15]. reinforce the message that not all competencies must be addressed in every course.

The faculty began by developing the course syllabi for the global health courses (See Table 1 for an overview of the curriculum). The programmatic content covers the eight domains for global health citizens: global burden of disease; globalization of health and health care; social and environmental determinants of health; collaboration, partnering, and communication; ethics; professional practice; health equity and social justice; and sociocultural and political awareness [7]. A significant portion of the program specifically focuses on leadership and collaboration which are required to meet the challenges of clinical practice or research in diverse cultures with vulnerable and underrepresented populations. Additionally, the course addresses the challenges that may arise when researchers attempt to establish research protocols in developing countries using research standards from developed countries [17]. Conflicts in cultural practices, misunderstandings of informed consent, variation in communication styles, and differences in literacy levels represent only a few of the challenges to overcome during the collaborative process when working or conducting research with low resource countries.

**Table 1:** Overview of Global Health Curriculum.

First Course	Social Determinants of Health in Low Resource Countries (3 credits)
Second Course	Leadership and Collaboration in Global Health (3 credits)
Elective	Students chooses one from a list of 10 pre-approved courses (3 credits)
Capstone Course	Field Experience in a Low Resource Country: Peru (3 credits)

Total Credits = 12 hours.

A supporting elective from foreign language, anthropology, women’s studies, or sociology is required. Utilizing current courses available throughout the university to meet the supporting elective requirement encourages a multidisciplinary approach, while providing a financial benefit by using currently available courses and eliminating the need for the School of Nursing to create another course.

For the capstone course, students participate in a month-long SA program in Peru where they immerse within the culture through their work, home environment, and language training. Strategically designed work opportunities provide practice with a sustainable, culturally appropriate health organization in the host country. The design of this field experience is supported by the research-based recommendations for international service-learning [9] and adheres to the Guidelines for Undergraduate Health-Related Programs Abroad established by The Forum on Education Abroad.

Once the course syllabi were developed, the faculty began the process of obtaining curricular approval. To facilitate the approval process, the lead faculty contacted other college and university departments who might have potentially, competing courses. This collaborative effort to connect with other departments allowed potential problems to be addressed prior to curriculum committee meetings. The lead faculty also presented to the Board of Trustees to receive approval for this “added value” certificate program for undergraduate students.

Time conflicts represented a minor barrier for offering the global health courses. Nursing students and other STEM disciplines tend to have rigid schedules and clinical practicums. Therefore, the global health courses were designed using both online and face-to-face class sessions, keeping in mind that students need to form connections during the early stages of the program to facilitate personal relationships with their peers and faculty. These relationships will be crucial when traveling abroad later in the program. Therefore, a face-to-face component of the program remains essential while using online components to ensure flexibility. Courses are scheduled during the evening hours to avoid time conflicts with nursing or STEM courses that are only offered during daytime hours.

Surprisingly, convincing nursing faculty that students could add additional courses to the rigid schedule presented an issue. Many of the nursing faculty expressed concerns that students would not be effectively able to manage the nursing curriculum while taking the additional global health courses. To address these concerns, an information session was offered during the Lead Teachers’ Meeting and the monthly departmental meeting in the School of Nursing. The nursing literature reports that students who engage in extracurricular activities during nursing school actually have increased professional effectiveness and improved self-confidence [18]. Wang and Shively [19] reported that students who participated in extracurricular activities demonstrated higher grade point averages (GPAs), higher retention and graduation rates, and higher standing within their programs. A study evaluating the impact of extracurricular activities and SA programs found that study abroad students took more credit hours and had higher GPAs than their domestic peers [20]. This study also found that SA students actually graduated sooner, which may result from greater academic planning. Therefore, if we take into account research-based findings, motivated students who engage in the global health program have a high probability of successfully completing the program when coupled with realistic advisement and appropriate academic planning.

## Implementation

Following curricular approval at the department, college, and university level, the first global health course began in fall of 2015. This course utilized a hybrid format (a combination of online and face-to-face class sessions) to allow for greater flexibility for students enrolled in programs with rigid schedules. This first course introduced students to the social determinants of health with a clear global focus on low resource countries. The focus on low resource countries provided a unique perspective that other university courses do not offer. Course topics included a historical perspective of global health, ethical issues of global healthcare delivery, economic factors, diseases of poverty, environmental health, traditional medicine, violence associated with conflicts of war, and lifespan issues of women, infants, children, and older adults. Assignments associated with this course included online exams, discussion boards, blogs, and a written paper. The global health blogs allowed students to take a topic of interest and further develop the topic with additional information from outside sources and graphics. According to Bloom’s Taxonomy, creating represents the highest level of cognition. The blogs required students to synthesize information and create new perspectives or products of thinking. Furthermore, blogs are interactive devices that facilitate reflection and place social value on relevant topics of interest [21] while increasing awareness of public health problems [22]. The written paper required the students to choose a prevalent health problem in a low resource country and examine the social, economic, environmental, political, and cultural factors that influence the disease process. The culmination of the assignment required 2–3 research-based strategies to address the health problem in a culturally appropriate manner.

The second global health course in the spring of 2016 utilized a similar hybrid format, but class sessions met in a virtual classroom. The class alternated between synchronous and asynchronous sessions to facilitate flexibility. Synchronous courses facilitate participation and motivation compared to asynchronous courses that allow more time for reflection of complex topics and the processing of cognitive information [23]. Course topics included cultural characteristics of groups, effective leadership strategies, cross-cultural communication strategies, teaching for low literacy populations, ethical issues of research, global planning, capacity building, and health care systems of low resource settings. Students were introduced to cultural care theories during the first course, yet every lecture emphasized the salient points of transcultural care skills and cultural humility. Several nurses with established reputations for global work were guest speakers throughout the semester. Each global expert provided current, real-world examples of successful health programs in low resource countries such as Peru, Cambodia, and Guatemala. Assignments associated with this course included online exams, discussion boards, group presentations, and a final written paper. The group presentation and written paper focused on collaboration with agencies working in low resource countries and the need for research-based, culturally appropriate inventions to address health issues. Throughout this course, the emphasis remained on the need to work collaboratively with local people of the host country, the relevance of cultural context for each unique setting, the intent of developing sustainable programs, and the need to empower the people of the community.

The inaugural, capstone course of the GHCP took place in the summer of 2016. Two faculty members and 11 students spent one month in Ollantaytambo, Peru. During this time, students and faculty lived with host families and attended approximately 10 hours of Spanish lessons per week. Approximately two days per week were spent working directly with a local non-governmental organization (NGO) in remote villages in the Peruvian Andes Mountains. These high altitude (2800 to 4100 meters above sea level) villages lack healthcare services, have high rates of poverty, and remain isolated from government services. In response to a request from the local NGO, the students had the unique opportunity to engage in data collection for a research study related to anemia; thus facilitating their introduction and participation in international research at the undergraduate level. The anemia screening conducted during this study established clear evidence of a severe public health problem with over 47% of participants being anemic [24]. Each week, students also attended approximately six hours of lecture to learn about the political dynamics, education levels, healthcare systems, and economic factors that influence healthcare in Peru. Additional lectures addressed ethnomedicine and cultural practices that were common among the indigenous Quechua people. Cultural excursions to archeological sites, such as Machu Picchu, were part of the course as well. Assignments for this course included reflection and development of teaching materials for the Quechua communities in the isolated villages. A repeat of the course in 2018 utilized assignments focused on strategic analysis, including a SWOT (strengths, weaknesses, opportunities, and threats) analysis of the community [16].

## Evaluation

Process level evaluation of individual courses was completed through student evaluations, which demonstrated a high level of satisfaction. End-of-course evaluations were rated on a scale of 1–5, with five being the highest level of satisfaction. Each evaluation score compared the overall mean score for the global health course with other nursing courses at the same level within the same discipline (SL/SD), and scores for courses of the same discipline (SD). Table 2 provides scores for each course and compares the scores to SL/SD and SD courses. Various departments across the university provide the supporting elective requirement courses; therefore, the end-of-course evaluations for those courses are not available. Clearly, students felt that the global health courses met the established objectives at or above the level of nursing courses for the same level and same discipline. Student self-perceptions revealed they felt better prepared to apply course content to culturally diverse populations than same level or same discipline courses after participating in the SA, capstone course.

**Table 2:** End-of-Course Evaluation Scores.

Evaluation criteria	Mean score for course	Mean score for SL/SD	Mean score for SD

Extent the course objectives were met	4.7*	4.6	4.6
	4.7**	4.5	4.6
	5***	4.5	4.6
Apply course content to culturally diverse populations	4.89*	4.45	4.33
	5**	4.3	4.4
	5***	4.3	4.5
Apply knowledge to solve health problems	4.67*	4.68	4.49
	4.8**	4.5	4.6
	5***	4.5	4.6

* First global health course. ** Second global health course. *** Study abroad, capstone course. SL/SD = course at the same level within the same discipline. SD = course within the same discipline.

Content level evaluation of the program focused on three student learning outcomes: 1) knowledge of social determinants of health, 2) communication skills for effective collaboration, and 3) an attitude of cultural humility. Knowledge of the social determinants of health were evaluated based on written papers discussing a global health problem with potential interventions to improve health outcomes associated with the topic. Communication skills for effective collaboration were evaluated based on written papers and a group presentation demonstrating communication skills for collaborative planning with host organizations. Communication skills were further evaluated at the outcome level through the development of a health promotion deliverable developed in conjunction with the host partner. Last, an attitude of cultural humility was evaluated through critical reflection and self-awareness with students completing a self-reflection journal. Each assignment was evaluated and scored based on a rubric.

The future employers of the GHCP graduates will eventually provide the true impact level of evaluation for the program. This inaugural class of the GHCP graduated in 2018. Employer surveys will be sent approximately six months after students graduate and begin working. The employer surveys will ask specific questions about the graduates’ ability to demonstrate competencies associated with global citizenry and provide culturally appropriate care with diverse populations.

## Recommendations for Future Global Health Programs

Faculty concerns related to course overload for nursing students or other STEM students should be acknowledged, addressed initially, and ongoing throughout the implementation of a new program. Preconceived ideas about additional course workload and SA experiences need to be dispelled through evidence-based, educational sessions. Faculty must recognize that research has demonstrated that extracurricular activities and SA may increase academic performance [20]. Adding more SA courses during the freshman and sophomore year may lead to improved four-year graduation rates according to Xu et al [20]. Furthermore, it is important to share with faculty the products of student experiences, such as reflective journals, blogs, research posters, presentations, or publications that may result from the global health courses or SA experience.

Recognition of time constraints is essential in planning the course format of a GHCP. Fully online programs have great appeal and may serve well to reduce barriers related to scheduling conflicts, but this must be balanced with the optimum strategies for learning and socialization. Synchronous e-learning allows for varied communication strategies and relationship building through social interaction [23]. This is particularly important for building student-to-student and student-to-faculty relationships prior to being immersed in a new culture for four weeks. Asynchronous e-learning facilitates learning content and allows more time for reflection on complex issues [23]. The appropriate mix of e-learning formats must be considered based on the expected program outcomes.

Capacity building implies an unequal relationship of power and knowledge between high resource and lower resource countries. Health professions must focus on shared knowledge and the bi-directional flow of knowledge between countries [5,6,25]. Knowledge transfer allows for the creation of new knowledge through education and research with a focus on meeting the needs of the information user. Furthermore, the concept of reverse innovation allows the shared knowledge gained in low resource countries to address health disparities in high resource countries, such as the US or a similar high resource country. Completing courses or programs in global health facilitates an awareness and understanding of the social determinants that affect health outcomes across geographical, economic, political, and cultural boundaries. This awareness supports utilization of appropriate resources to address health disparities here “at home” when students choose to work in the US. Therefore, it is crucial to design the fieldwork of a global program in a manner to support this bi-directional flow of information and research.

Additionally, new global health programs must place a high value on interprofessional education [7] to avoid the “profession-centrism” that develops when nursing or physicians work in silos [14] When students from various disciplines learn together, they recognize and value the unique contributions of each discipline. As partners, they form teams to address the multitude of complex social determinants that affect health. Developing a true interprofessional program requires a great deal of collaboration and mutual respect among colleagues from different disciplines within a university. Consistent and clear communication is key to this collaborative process from the initial stages of program design through implementation and ending with evaluation of program outcomes.

## Conclusion

The United Nations (UN) Sustainable Development Goal #10 addresses the need to “reduce inequalities within and among countries [26].” The UN further recognizes the need to go beyond focusing only on economic conditions, but also to address social and environmental issues that influence inequality. Nursing and other health professionals must be educated about these issues at the global level. While Premji and Hatfield [5] have so eloquently called for a new paradigm, “One World, One Health,” health professionals must step to the forefront and prepare the next generation of healthcare leaders about health inequalities of our global community partners. Ultimately, this may effectively allow us to address the health inequalities within our own nation.
